# Breeding Beyond Monoculture: Putting the “Intercrop” Into Crops

**DOI:** 10.3389/fpls.2021.734167

**Published:** 2021-11-18

**Authors:** Peter M. Bourke, Jochem B. Evers, Piter Bijma, Dirk F. van Apeldoorn, Marinus J. M. Smulders, Thomas W. Kuyper, Liesje Mommer, Guusje Bonnema

**Affiliations:** ^1^Plant Breeding, Wageningen University & Research, Wageningen, Netherlands; ^2^Centre for Crops Systems Analysis, Wageningen University & Research, Wageningen, Netherlands; ^3^Animal Breeding and Genomics, Wageningen University & Research, Wageningen, Netherlands; ^4^Farming Systems Ecology Group, Wageningen University & Research, Wageningen, Netherlands; ^5^Field Crops, Wageningen University & Research, Lelystad, Netherlands; ^6^Soil Biology, Wageningen University & Research, Wageningen, Netherlands; ^7^Plant Ecology and Nature Conservation, Wageningen University & Research, Wageningen, Netherlands

**Keywords:** intercropping, plant breeding, functional–structural plant modeling, indirect genetic effects, plant–plant interactions, mycorrhiza, plasticity

## Abstract

Intercropping is both a well-established and yet novel agricultural practice, depending on one’s perspective. Such perspectives are principally governed by geographic location and whether monocultural practices predominate. Given the negative environmental effects of monoculture agriculture (loss of biodiversity, reliance on non-renewable inputs, soil degradation, etc.), there has been a renewed interest in cropping systems that can reduce the impact of modern agriculture while maintaining (or even increasing) yields. Intercropping is one of the most promising practices in this regard, yet faces a multitude of challenges if it is to compete with and ultimately replace the prevailing monocultural norm. These challenges include the necessity for more complex agricultural designs in space and time, bespoke machinery, and adapted crop cultivars. Plant breeding for monocultures has focused on maximizing yield in single-species stands, leading to highly productive yet specialized genotypes. However, indications suggest that these genotypes are not the best adapted to intercropping systems. Re-designing breeding programs to accommodate inter-specific interactions and compatibilities, with potentially multiple different intercropping partners, is certainly challenging, but recent technological advances offer novel solutions. We identify a number of such technology-driven directions, either ideotype-driven (i.e., “trait-based” breeding) or quantitative genetics-driven (i.e., “product-based” breeding). For ideotype breeding, plant growth modeling can help predict plant traits that affect both inter- and intraspecific interactions and their influence on crop performance. Quantitative breeding approaches, on the other hand, estimate breeding values of component crops without necessarily understanding the underlying mechanisms. We argue that a combined approach, for example, integrating plant growth modeling with genomic-assisted selection and indirect genetic effects, may offer the best chance to bridge the gap between current monoculture breeding programs and the more integrated and diverse breeding programs of the future.

## Introduction

Agriculture is at a crossroads. On the one hand, industrialized agricultural systems have delivered high yields of staple crops, achieved through a combination of chemical inputs, improved varieties, mechanization and large-scale agribusiness farms(Tilman et al., 2002). Despite its successes, modern agriculture is a system that is clearly out of balance and one that has led to widespread problems for soil, water, biodiversity, climate, and health (Steffen et al., 2015). One of the solutions proposed is to re-align our agricultural system with natural processes and cycles through the re-diversification of our cropping systems (Vandermeer, 1992; Brooker et al., 2015). Such diversified cropping systems (alternatively referred to as intercropping, mixed cropping, or polyculture) are already widely deployed in smaller-scale farming operations in many parts of the world [particularly in Latin America, Africa but also China (Brooker et al., 2015)].

The simultaneous cultivation of more than a single crop, including a diversity of genotypes of a single-crop species (Smithson and Lenné, 1996; Chateil et al., 2013), can lead to higher yields and increased yield stability and food security (Raseduzzaman and Jensen, 2017) of critical importance in low-input, often small-scale agricultural systems. There is an urgent need to investigate how more diverse cropping systems can be applied on larger spatial scales (Feike et al., 2012; Bybee-Finley and Ryan, 2018), particularly in the context of the current set of Sustainable Development Goals and the sustainable intensification needed to achieve them (Struik and Kuyper, 2017). Up-scaling of crop mixtures will require a re-designing of the technology currently employed in large-scale agricultural systems.

Among these technical means, one of the key components is modern improved varieties, as these have significantly contributed to the increase in yield and other important agronomic and economical traits. One example is the high-yielding modern dwarf varieties of wheat and rice that were first deployed during the Green Revolution of the 1950s and 1960s (Evenson and Gollin, 2003). Most modern varieties are bred specifically for monoculture, where a single genotype is grown (spatial monoculture). In our definition, this is irrespective of what crop was grown in preceding or subsequent seasons. However, modern varieties bred for monoculture are unlikely to be the best adapted genotypes for diverse cropping systems (Hamblin and Zimmermann, 1986; Hill, 1990; O’Leary and Smith, 1999; Brooker et al., 2015; Annicchiarico et al., 2019). Current breeding strategies focusing on the selection of the best performing genotypes in pure stands have overlooked the benefits of positive inter- and intraspecific interactions between crops or genotypes. Breeding practices and protocols are geared toward breeding for pure stands, ignoring the potential impact of trait variation of a companion crop on a plant’s performance.

Biodiversity is one of the key factors underpinning ecosystem functioning (Tilman et al., 2014; Weisser et al., 2017; Leclère et al., 2020) and is a priority within the United Nation’s sustainable development goal 15 (sdgs.un.org). Biodiversity is the combination of ecosystem diversity, species diversity, and genetic diversity within species. While most ecological studies have focused on the importance of species diversity for ecosystem functioning, the erosion of crop genetic diversity is often seen as a more critical issue (Hajjar et al., 2008) in agriculture. Examples include genetic bottlenecks arising from breeding activities (Louwaars, 2018) or the replacement of farmers’ landrace varieties with modern cultivars (F.A.O., 2019). Over the last half century, there has been a general trend toward reduced diversity in cropping systems both across and within species, with a concentration of agricultural production from an increasingly small number of key or staple species (Dawson et al., 2019). Intercropping provides an opportunity to re-diversify agricultural systems on many levels: increased diversity of crop species within land parcels, increased diversity within a crop species across cropping systems, and increased non-crop diversity within the agricultural landscape of wild species (Koricheva and Hayes, 2018; Beillouin et al., 2019).

Although there are clearly many reasons why plant breeding programs *should* accommodate diversity (Østergård et al., 2009; Lammerts Van Bueren et al., 2018), in practice many modern plant breeding programs are commercial operations that make breeding decisions based on economic justifications. If plant breeding companies are to begin to breed for more diversified agricultural systems, they will do so only when a number of economic justifications are already satisfied. These could include (1) forecasts on which crop combinations will primarily be used by farmers and growers in the future and at what scale this will occur, (2) the market potential for an adapted cultivar for intercropping over a standard cultivar, and (3) the relative efficiency versus costs of breeding under mixed stand conditions compared with pure stands. There is an urgent need to explore these questions together with breeders, some of whom already recognize the benefits of diversification but do not yet consider there to be a need to actively begin breeding for such systems (Dawson et al., 2019). The reasons for this could be economic as listed above, but could also be practical as there is currently little guidance or expertise on how breeding for intercropping should be performed.

Other authors have highlighted the issue of breeder engagement and suggest that farmers should be involved in the process of breeding for intercropping through participatory plant breeding programs (Annicchiarico et al., 2019). For now, we assume that breeders are ready and willing to take up the challenge. We therefore focus primarily on the challenges faced by breeders in developing new variety combinations and the potential of modern computational methods for use in more diverse breeding programs. We identify a number of breeding directions for intercrop performance. We firstly explore the idea of “trait-based breeding,” taking inspiration from the results of plant growth modeling and ecological theory to provide specific trait-based breeding targets to define a crop ideotype. A complementary breeding approach is what might be termed “product-based” breeding, in which a statistical black-box approach is used to optimize the system rather than breeding by design toward an ideotype. Quantitative breeding approaches are already widely used in animal and plant breeding programs, for example, in the use of genomic prediction models (Meuwissen et al., 2001). In intercrop breeding, genomic prediction could reduce the need for extensive phenotyping (Annicchiarico et al., 2019) while potentially achieving greater genetic gains than traditional phenotypic selection programs (Bančič et al., 2021). We propose that an integrated framework that combines information from both approaches could lead to both continual genetic improvement and the prediction of breakthrough trait combinations.

To be able to discuss the integration of these mechanisms into breeding for intercropping, we first review our current understanding of the biological mechanisms that can lead to improved performance of crop mixtures over pure stands.

## Biological Mechanisms in Intercropping

### Eco-Physiological Mechanisms Underlying Crop Mixture Performance

Growing mixtures of species or genotypes is often more productive than pure stands (Bedoussac et al., 2015; Brooker et al., 2015), demonstrating higher nutrient efficiencies and increased biocontrol, leading to more sustainable agricultural systems (Boudreau, 2013; Li et al., 2014). However, relatively little work has been done to explore the potential of crop mixtures for modern agriculture, even though this potential has been shown for mixtures of species (Yu et al., 2015; Fletcher et al., 2016; Juventia et al., 2021) and genotypes (Tooker and Frank, 2012; Sapoukhina et al., 2013; Ditzler et al., 2021). Here, we consider a crop mixture to include both mixtures of genotypes of a single species, or mixtures of different species, encompassing a range of possible spatial and temporal arrangements (Brooker et al., 2015).

Recent research has started to focus on the mechanisms that explain the increased performance and efficiency of mixed-species systems (Stomph et al., 2020). One of the reasons crop mixtures show these benefits can be traced back to the way plants of different species compete for resources. Relaxation of competition between species due to spatial or temporal complementarity in resource uptake is a strong determinant of mixture performance and efficiency (Yu et al., 2015; Li et al., 2020). For instance, differences in root growth or root architectural characteristics between species growing together may lead to complementary uptake of water or nutrients, when the root systems are (partly) spatially or temporally separated (Henry et al., 2010; Postma and Lynch, 2012). Similarly, differences in shoot architecture and photosynthetic efficiency can result in complementarity in light capture and light use efficiency (Stomph et al., 2020), especially when the component species are not sown or harvested simultaneously (Yu et al., 2015).

Further mechanisms underlying high performance and efficiency of mixtures relate to a reduction in the prevalence of weeds and diseases in mixed systems. In theory, high weed suppression by one of the component species in a mixture may lead to improved performance of the other, leading to more productive and resource-efficient crop systems. Ideally, weed suppression should occur without incurring negative competitive effects on the component crop species, replacing weed biomass with crop biomass. Enhanced weed suppression in crop mixtures does occur (Stomph et al., 2020), while ecological studies have also demonstrated that invading species such as weeds have less opportunity to invade diverse plant communities compared to monocultures (Van Ruijven et al., 2003). Disease incidence can be reduced drastically in crop mixtures (Stukenbrock and McDonald, 2008; Boudreau, 2013; Wuest et al., 2021) for both leaf and soil-borne diseases. Disease suppression in mixtures has been attributed to host dilution, allelopathy, and microclimate effects, and depending on the design of the mixture, also physical barrier effects (Ampt et al., 2019).

Importantly, plant traits that may provide benefits in one type of mixed-crop system may not be relevant for high performance in another. Mixed-crop systems come at many different levels of temporal and spatial species segregation (Ditzler et al., 2021). For example in fully mixed designs, the component species are fully mixed within the crop rows. There are also a range of strip cropping systems (Van Oort et al., 2020). Narrow strip systems maximize interspecific interaction but rule out mechanical management (strips of one or two rows per species alternating). Alternatively, wide-strip systems show very little interspecific interaction but provide other benefits, such as complementary insect populations that improve pollination and herbivore reduction, or beneficial microclimate (alley cropping). It is therefore likely that crop genotypes with a particular set of traits may only show the typical mixed-crop benefits for a subset of mixture designs.

### Deciphering Interactions in Intercropping

Central to the topic of intercropping is the extent to which interactions between plants will affect overall intercrop performance. A better understanding of these interactions can lead to insights into how best to design an intercrop system and may also provide leads for breeding. However, the literature on such interactions often contains discipline-specific terminology and classifications and requires some “deciphering” for the non-specialist reader.

In much of the general agronomic literature on intercropping, three types of plant–plant interactions are mentioned: competition, complementarity, and facilitation (Li et al., 2013; Bedoussac et al., 2015). Competition is generally framed as an undesirable interaction, leading to a negative impact on the performance of one or both species. Complementarity and facilitation, on the other hand, generate positive effects on intercrop performances (Li et al., 2014; Barry et al., 2019). However, competition effects in intercrops may also provide benefits, at least temporarily. For example, the combination of a cereal and a legume can benefit from the direct competition for soil inorganic N between the species, forcing the legume, as weaker competitor, to invest more in its rhizobial symbionts to supply its nitrogen needs (Jensen, 1996). This results in an emergent behavior of the mixed-cropping system to become more N-efficient (i.e., increased production per unit of N input), an effect also known as N-sparing (Giller, 2001). Therefore, in this situation, competition ultimately leads to facilitation and complementarity.

**Figure 1:**
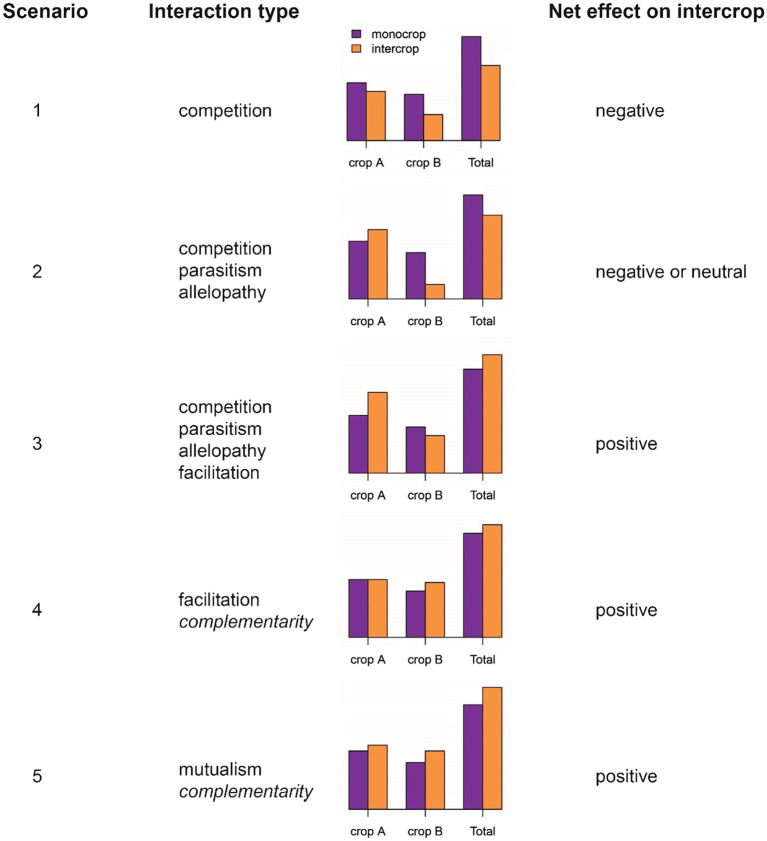
Types of inter-specific plant-plant interactions. Five scenarios are depicted with contrasting net effects on intercrop performance (for example, the economic value of the combination of crops). “Interaction type” includes the terms used to describe the nature of the interaction, summarized from Dudley (2015). Complementarity is omitted from Dudley’s classification.

More nuanced classifications of plant–plant interactions identify both costs and benefits to each component species and distinguish between inter- and intraspecific interactions (Dudley, 2015; Subrahmaniam et al., 2018). Identifying costs and benefits provides a sound classification framework. But from an agronomic and breeding perspective, one is most interested to know whether the net effect of a specific intercrop (effect=benefit−cost) is positive, neutral or negative to the overall performance metric to be maximized or improved. To illustrate this, we present five possible scenarios that demonstrate different cost/benefit relationships between a pair of intercrop partners in Figure 1. In the first scenario, competition proves detrimental to both parties, with a negative net effect. Alternatively, one crop may benefit at the other’s expense, resulting in either a neutral or negative net effect, depending on the magnitude of competition and the relative value of the component crops (scenario 2; Figure 1). In such circumstances, parasitism or allelopathy may be involved in the interaction, although they suggest particular life-cycle strategies or mechanisms that go beyond simple “competition.” In the third scenario, the benefit enjoyed by one crop exceeds the cost paid by the second crop, resulting in a positive net effect. Although competition still occurs (at least from the perspective of the crop paying the price), such an interaction could also be termed “facilitation,” enabling a superior overall performance in combination (e.g., in a legume-cereal combination). Facilitation may also occur at no cost to the enabling partner (scenario 4; Figure 1). In the most ideal scenario, facilitation may be reciprocal, i.e., in a “mutualistic” interaction (scenario 5; Figure 1). However, in order to quantify costs and benefits, one needs information on pure stand performance. This is certainly of scientific interest, but it is unlikely that future intercrop breeding will involve calculations of costs and benefits (unless perhaps through *in silico* simulation). On the other hand, it is straightforward to assign economic weights to component crops in a joint crop analysis, an approach presented in more detail below (*cf*. section “The Direction of Selection”).

In the ecological literature, the concept of “niche differentiation” is generally used to describe the process whereby species have evolved to avoid each other’s specific niches (Zuppinger-Dingley et al., 2014; Meilhac et al., 2020). Niche differentiation leads to the avoidance of direct competition by expanding the range of microniches and ultimately leads to greater overall productivity of the assemblage (Gathumbi et al., 2002; Li et al., 2007; Ndufa et al., 2009; Mueller et al., 2013). Ecologists also frequently partition biodiversity effects into *complementarity* versus *selection* effects (Loreau and Hector, 2001). Positive selection effects in a mixture occur when highly productive species in monoculture also dominate in the mixture, and positive complementarity effects occur when species’ yields in the mixture are on average greater than expected from their yields in monoculture, weighted by their relative abundance in the mixture (Loreau and Hector, 2001). The complementarity effect is the difference between the net biodiversity effect (observed yield of the mixture minus the yield of the mixture expected without selection and complementarity effects) and the selection effect. It covers a range of plant–plant interactions including niche partitioning and facilitation (Barry et al., 2019). Thus, while complementarity is often presented as being distinct from facilitation in the general intercropping literature, the terms are not considered exclusive in the ecological additive-partitioning of biodiversity effects. The interested reader is directed to the review of Barry et al. (2019) that highlights this confusion and suggests how the study of complementarity might be better approached in future research (Barry et al., 2019).

### Plant Plasticity

Plasticity in plant traits (the ability of a plant to morphologically adapt its phenotype to a particular environment) can help maintain a balance between intercrop partners through niche differentiation, which may ultimately lead to over-yielding. However, it potentially complicates the definition of an ideotype in a more variable growing environment such as an intercrop. Many types of intercrops typically have some degree of spatial heterogeneity due to differences between conditions experienced by individual plants. If plants are plastic, they can tailor their growth and development to the resources locally available (e.g., Zhu et al., 2016). Plants in mixed-cropping systems encounter different local environments above and below ground due to their diverse neighboring plants. As a consequence, a plant phenotype is the result of these variable local phenotypic responses, maximizing resource uptake and potentially leading to higher overall performance. On the other hand, plasticity comes at a cost. Plants with limited resources may expend unnecessary resources in trying to acquire more resources, e.g., by stem elongation that may be detrimental to overall crop performance. In monoculture cultivation, such plastic responses (e.g., unwanted side-shoot development) are partly controlled through planting density. In an intercrop, that means of control may no longer be effective. Plasticity may also lead to certain non-uniformity in a crop that can be detrimental to marketable yield. Plasticity may thus help to improve intercrop performance, but may also reduce it. Plastic responses to acquire extra available resources are beneficial, but plastic responses to escape adverse conditions or those that reduce yields may ultimately be detrimental for whole crop performance. Breeding may therefore be needed to increase plasticity for some traits (e.g., those involved in competition) but not for others (e.g., those involved in marketable yields).

### Using Functional–Structural Plant Modeling for Intercrop Breeding

Most studies on crop mixtures rely on field experiments and occasionally detailed pot experiments to understand and/or predict performance of intercrops. Such experiments provide very useful information on plant behavior in mixed systems and its consequences for overall crop performance. However, they are limited in the extent to which plant traits can be changed or manipulated, in the number of scenarios that can be tested and in the level of detail in the data they can generate. Simulation modeling has been a useful tool in complementing experimental work on species mixtures as well as in informing it (Gaudio et al., 2019).

An approach to capture mixture behavior in simulation is to adapt crop models that have been developed to simulate pure crops and modify them to represent crop mixtures (Corre-Hellou et al., 2009; Chimonyo et al., 2016). This approach is useful when representing full mixtures with little or no spatial heterogeneity. However, species mixtures with a distinct spatial arrangement, such as strip intercrops, cannot be represented satisfactorily in such models. This has led to the development of models that capture strip arrangements as combinations of small pure stands, still using traditional crop modeling approaches (Gou et al., 2017; Van Oort et al., 2020). Approaches relying on traditional crop modeling concepts do not allow the exploration of combinations of species phenotypes (development, physiology, architecture) for crop design optimization (e.g., varying the level of plant clustering in strips, population densities, amount of temporal overlap). This is because (1) the phenotype of the species used in simulation is captured in a relatively small set of parameters in such models and (2) the degree to which plant arrangement can be altered is limited.

**Figure 2:**
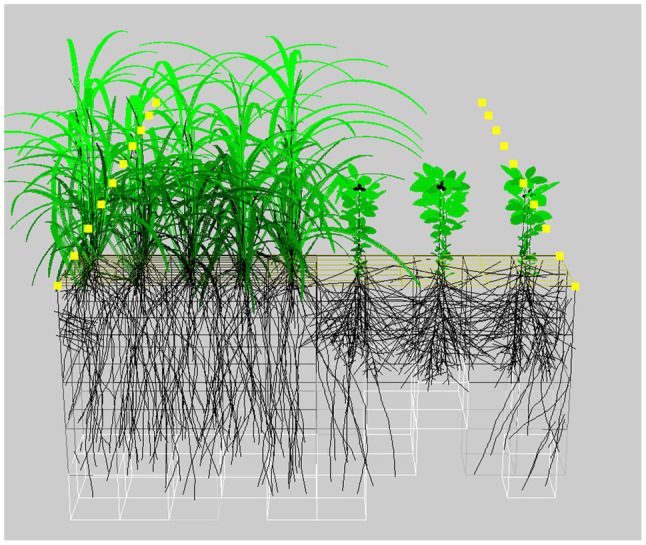
Example of an FSP simulation of a relay cereal-legume system at 52days after sowing. The brightness of the leaves corresponds to the leaf light capture. The boxes surrounding the roots represent the soil explored by the roots. Image created using the GroIMP modeling platform (Hemmerling et al., 2008).

The functional–structural plant (FSP) modeling approach (Godin and Sinoquet, 2005; Evers et al., 2018) does not have these drawbacks. In FSP models, plant development, growth and architecture are simulated in 3D over time and are governed by the effects of competition for capture of resources such as light, water, and nutrients (Figure 2). Originally developed to represent plant development realistically (Prusinkiewicz and Lindenmayer, 2012) and not to predict crop performance, plant traits such as leaf size and angle, stem length, and root branching are explicitly captured in FSP models. This makes FSP modeling ideally suited to explore the relationships between plant traits, plant arrangement, and performance. This has been done successfully for leaf traits in tomato pure stands (Sarlikioti et al., 2011) and wheat–pea mixtures (Barillot et al., 2014) as well as for root traits in single bean plants (Rangarajan et al., 2018).

These examples demonstrate the possibilities of FSP modeling to help breeding for diversity. However, to truly arrive at an optimized combination of species phenotypes and plant arrangement, FSP models should capture both above- and belowground processes in sufficient detail (Faverjon et al., 2019; De Vries et al., 2021). Plant growth in any crop type is restricted by the most limiting resource, and thus, the capture and use of those resources needs to be well represented to model plant growth (Evers et al., 2019). Ultimately, FSP models can be applied to explore the interaction between species traits and intercrop plant arrangement. Architectural ideotypes, complementary in resource use, could thus be determined as optimized phenotypes for mixture designs. A clear description of these architectural ideotypes could inform breeding programs while alleviating the challenge of having to test large numbers of genotypes and crop designs.

### Interactions In the Soil Involving Mycorrhiza

In breeding, most attention is focused on above-ground plant traits (with the obvious exception of root crops). In contrast to above-ground traits, our understanding of variation in root traits has lagged behind (Faget et al., 2013; Weemstra et al., 2016). However, our view of roots has recently been transformed (Bergmann et al., 2020; Laughlin et al., 2021), with a large proportion of root trait variation being explained by the propensity of a plant to form a symbiotic association with mycorrhizal fungi (beneficial associations between certain root-inhabiting fungi and plant roots). Among crop species, almost all crop plants form such mycorrhizas, contributing to enhanced uptake of nutrients of limited mobility (especially phosphate, also zinc and copper) and water, and increasing resistance or tolerance to biotic and abiotic stresses. Crop species differ in the extent to which they depend on and benefit from this mycorrhizal symbiosis. Variation in plant response to mycorrhiza has also been reported for many crops (Kuyper et al., 2021).

It is likely that modern agronomic practices (continuous monocropping, high fertilizer use, fungicide use, tillage, bare fallow in the winter season) have selected against mycorrhizal fungi with larger benefits to plants (Verbruggen and Kiers, 2010). It has also been hypothesized that plant breeding under these conditions has resulted in plants with lower benefits from the mycorrhizal symbiosis (Hetrick et al., 1992). Intercropping systems are often characterized by lower fertilizer and fungicide levels, less soil disturbance, and higher plant diversity and cover. Intercropping should therefore shift the current selection of mycorrhizal fungal species with ruderal life styles and limited plant benefit toward species that form more beneficial associations.

It is widely accepted that mycorrhizal fungi show little or no selectivity with regard to the plant species with which they associate. Consequently, the mycorrhizal mycelium in soil consists of a network through which plants are connected, known as common mycorrhizal networks (CMN). These CMNs have been shown to underlie the overyielding of plant species mixtures (Walder et al., 2012) or variety mixtures (Wang et al., 2020). Although the extent of plant and fungal control over movement of carbon and nutrients through such CMNs is poorly known, it is possible that such CMNs reduce rather than amplify competition, resulting in a negative selection effect and a positive complementarity effect. If this is a general pattern, it could imply that plant breeding for intercropping should ensure plants be sufficiently promiscuous, associating with a diversity of mycorrhizal fungi to promote the development of CMN and their resulting overyielding benefits.

The issue of breeding for mycorrhizal promiscuity or selectivity has received no attention to date, contrary to nitrogen-fixing bacteria (rhizobia) in soya bean (*Glycine max*). Soya bean cultivars have been bred in Africa that were able to associate with indigenous rhizobia, thereby foregoing the need for inoculation. Alternatively, soya bean cultivars have been bred in the United States that very specifically associate with a limited number of rhizobial strains (Giller, 2001, p. 155–157). This points to a genetic basis for symbiont selectivity, and the merits and disadvantages of breeding to modify symbiont selectivity should be further investigated.

## Breeding Directions for Intercrop Performance

### Ideotype Breeding

In plant and animal breeding, ideotypes have been used to describe a conceptual direction toward which a breeding program can aim. An ideotype describes an ideal or hypothetical phenotype that is expected to maximize performance (usually yield) under a particular set of growing conditions. Particularly in plant breeding, individual performance of plants grown together is of secondary importance to their collective performance (Weiner, 2019). When originally proposed, the ideotype concept was used to describe an ideal wheat plant (*Triticum aestivum*): weakly competing and tolerant of both high planting densities and high soil fertility (Donald, 1968). Donald’s wheat ideotype most likely benefitted from hindsight: dwarf rice and wheat varieties providing the inspiration for the broader concept of ideotype breeding (Rasmusson, 1987). The idea of breeding for an idealized individual, one that may demonstrate poor individual fitness under natural selection but leads to superior collective performance, has remained a powerful concept, particularly for plant breeders. In some crops like rice, there is evidence to suggest that following an ideotype breeding approach has led to higher genetic gains for yield than would have been expected under selection for yield alone (Peng et al., 2008).

Ideotype breeding focuses primarily on defining breeding targets for traits which are thought to contribute to higher crop performance, in a real or hypothetical environment (Donald, 1981). Most ideotypes assume a monoculture cropping system, where a plant experiences a neighborhood of identical genotypes. However, the definition of ideotype does not preclude cropping systems that involve non-kin neighbors. Indeed, the term “ideomix” has been coined to extend the ideotype concept to plant mixtures (Litrico and Violle, 2015).

An intercrop ideotype would ideally include a range of positive interaction effects that optimize collective performance. While a single wheat genotype can be selected to poorly compete with its conspecifics in a monoculture stand, it is less clear what sort of interactions should be selected for among intercrop partners, particularly given the dynamic and inter-dependent nature of these interactions. An increasingly detailed description of favorable interaction effects is being compiled, although it remains context-, crop-, and experiment-specific in many cases (Brooker et al., 2021). Efforts to generate *in silico* ideotypes are providing novel insights (Louarn et al., 2020), but still require confirmation of their ability to predict as-yet unidentified traits with significant agronomic impact. As we develop greater insight into the mechanisms involved in intercrop performance, it is likely that more detailed crop combination-specific intercrop ideotypes will emerge.

### A Quantitative Genetic Approach to Breeding for Intercropping

Many relevant traits in plant production are quantitative and affected by many genes. This is particularly the case for yield. For such traits, quantitative genetics provides a powerful and mathematically explicit framework for genetic improvement. Developments in genomic prediction (Daetwyler et al., 2013) and indirect genetic effect (IGE; Bijma, 2014) make this approach very suitable for intercropping.

In most cases, the choice of a production system will precede the genetic improvement for that system, and the desired direction of genetic improvement follows from the properties of the production system. Hence, to discuss genetic improvement in the context of intercropping, we will assume here that the crops that are grown together have already been chosen. Thus, we will focus on genetic improvement in an existing intercropping production system, where the two (or more) species are a given. Though the focus is on a system of two species, the concept generalizes to more than two. Moreover, we will focus on recurrent selection, for example, to improve the *per se* value of populations in outbreeding species or to ultimately deliver hybrid cultivars, such as reciprocal selection or topcross selection.

Genetic improvement for intercropping differs from breeding for monoculture only when the two species grown together impact each other and when this impact shows genetic variation. Without such impact, the optimum breeding direction will be the same as for monoculture, while the absence of genetic variation makes breeding futile. For this reason, IGEs that act between the two crops grown together are the key element that differentiate genetic improvement for intercropping from breeding for monoculture. The importance of IGEs for intercropping has also been recognized by other authors, most notably in the contribution of Wright (1985) although there the term “associate effect” was used. We describe here the steps of incorporating IGEs in a prediction model for an intercrop to provide a bridging link in the literature and offer a fresh perspective on Wright’s approach.

An IGE is a genetic effect of one individual on the trait values of another individual. Neighboring plants may, for example, impact each other’s growth rate and this impact may have a genetic basis. Traditionally, IGEs have been defined for individuals of the same population and thus species (Griffing, 1967; Moore et al., 1997; Muir, 2005). However, there is no conceptual difficulty to extend the IGE concept to interactions between species. When breeding for monoculture production, within-population IGEs are implicitly accounted for when selection occurs at the level of plots of a single genotype or of a family (Griffing, 1976). With intercropping, however, also between-population IGEs matter, and those require specific attention.

### The Direction of Selection

In an intercropping production system, interest is typically in the performance of the entire system, which may include the performance of both crops, say *M* and *F*, indicating maize (*Zea mays*) and faba bean (*Vicia faba*) which we use as an example. Because the relevant importance may differ between the two crops, we may specify a quantitative breeding objective, say *H*, which is a weighted (*w*) sum of the relevant phenotypes of each of the two crops,


$$H=w_{M}y_{M}+w_{F}y_{F}$$

where for the sake of example, $y_{M}$ represents the yield of maize and $y_{F}$ the yield of faba bean. More generally, $y_{M}$ and $y_{F}$ could be a combination of multiple traits of each of the two crops. When the goal is to increase profit of the entire system, the weights *w_M_* and *w_F_* would be partial derivatives of profit with respect to yield of maize and yield of faba bean, respectively, following basic principles of selection index theory (e.g., Smith et al., 1986). When interest is in only one of the two crops, for example, when the second crop is a rhizobial symbiont grown to increase yield of the first crop, one can simply set *w*_2_ to zero.

Maize yield will depend on the genes of maize (direct genetic effect, DGE), but may also be affected, *via* competition or facilitation, by genes of the faba bean. The latter represents a between-species IGE. The same applies to the yield of faba bean. Hence, in total we need to consider four quantitative genetic main effects: DGE of maize on yield of maize ( $G_{M,D}$ ), DGE of faba bean on yield of faba bean ( $G_{F,D}$ ), IGE of maize on yield of faba bean ( $G_{M,I}$ ), and IGE of faba bean on yield of maize ( $G_{F,I}$ ). There are also two inter-specific interaction effects, namely $G_{M,D}*G_{F,I}$ and $G_{F,D}*G_{M,I}$ (Sampoux et al., 2020). The previous equation for *H* can therefore be expressed more fully as


$$\begin{array}{l} H=w_{M}\left(\mu_{M}+G_{M,D}+G_{F,I}+G_{M,D}*G_{F,I}\right)+\\ \;\;\;\;w_{F}\left(\mu_{F}+G_{F,D}+G_{M,I}+G_{F,D}*G_{M,I}\right) \end{array}$$

where $\mu_{M}$ and $\mu_{F}$ are the mean contributions to *H* of maize and faba bean, respectively.

In a recurrent selection cycle where genotypes are randomly assembled, only the genetically additive parts of direct and indirect effects are inherited. Denoting the heritable component of *H* by *H_ADD_*, we have


$$H_{ADD}=w_{M}\left(A_{M,D}+A_{F,I}\right)+w_{F}\left(A_{F,D}+A_{M,I}\right)$$

where $A_{M,D}$ , $A_{F,I}$ , $A_{F,D}$ , and $A_{M,I}$ are the additive genetic values inherited in the next generation by offspring of candidates to selection for $G_{M,D}$ , $G_{F,I}$ , $G_{F,D}$ , and $G_{M,I}$ , respectively. Note that the genetic effects are indexed by the crop from which they originate, because this crop is the gene pool relevant for the improvement of the genetic main effect. For example, $G_{F,I}$ is the IGE due to faba bean on the yield of maize; improvement of $G_{F,I}$ requires breeding in faba bean, but will benefit maize yield.

A relatively larger variance of the interaction terms indicates a smaller narrow sense heritability. Hence, the magnitude of the interaction variance is relevant for the choice between a recurrent selection scheme vs. a general mixing ability scheme (e.g., Sampoux et al., 2020). Furthermore, when the aim is to develop a specific two-genotype combination (a “tandem” variety pair), the interaction between genotypes is of interest. Focusing on the additive part of the model, selection in maize would be for the selection index


HADD,M=wMAM,D+wFAM,I

while selection in faba bean would be for the index


$$H_{ADD,F}=w_{F}A_{F,D}+w_{M}A_{F,I}$$

Note that *H_ADD,M_* could be considered as a weighted general mixture ability of maize (respectively, *H_ADD,F_* for faba bean). If *w_M_*=*w_F_*=1, then *H_ADD,M_* is the general mixture ability of maize (Sampoux et al., 2020). The direct component of *H_ADD,M_* and *H_ADD,F_* will be expressed in the crop itself, while the indirect component will be expressed in the partner crop.

The total genetic variation that breeders can use for improvement of the intercropping system by recurrent selection is equal to the variance of *H_ADD_* (Wright, 1985; Bijma, 2011; Sampoux et al., 2020). For maize, this equals


$$\begin{array}{l} \mathrm{var}\left(H_{ADD,M}\right)=w_{M}^{2}\mathrm{var}\left(A_{M,D}\right)+w_{F}^{2}\mathrm{var}\left(A_{M,I}\right)+\\ 2w_{M}w_{F}\mathrm{cov}\left(A_{M,D},A_{M,I}\right) \end{array}$$

and for faba bean


$$\begin{array}{l} \mathrm{var}\left(H_{ADD,F}\right)=w_{F}^{2}\mathrm{var}\left(A_{F,D}\right)+w_{M}^{2}\mathrm{var}\left(A_{F,I}\right)+\\ 2w_{M}w_{F}\mathrm{cov}\left(A_{F,D},A_{F,I}\right) \end{array}$$

A trade-off due to competition will reduce this genetic variation. For example, if selection of maize for higher yield reduces yield of faba bean due to competition, then this will surface as a negative covariance between $A_{M,D}$ and $A_{M,I}$ . The absolute magnitude of this competition is measured by the size of this (negative) covariance. The deviations of the corresponding correlations from a value of −1 indicate the degree to which this trade-off can be circumvented by selection. Since genetic correlations are rarely equal to −1, the presence of a trade-off does not imply that simultaneous improvement of the yield of both crops is impossible; it merely means a slower rate of improvement. Moreover, these two correlations indicate that the total trade-off may originate from two different gene pools, and the strength of the trade-off may differ between the two gene pools. In other words, maize competing with faba bean does not imply faba bean competing with maize. In the absence of competition, positive covariances between DGE and IGE are possible, implying genetic variability for facilitation.

The relationship between direct and IGEs was also recently used to qualitatively classify the nature of interaction effects between intercrop partners (Haug et al., 2021). The authors developed an elegant classification system based on nine different potential combinations (either −/0/+ for the direct and indirect genetic effects) in a binary mixture, with terminology reminiscent of other plant–plant interaction classification systems previously mentioned (Dudley, 2015; Subrahmaniam et al., 2018). These provide a more intuitive understanding for breeders of the types of interaction effects that ultimately should be aimed at in breeding programs.

### Estimating Direct and Indirect Genetic Effects

Genetic improvement of the overall performance of an intercropping system requires estimates of the direct and indirect genetic components of *H_ADD,M_* and *H_ADD,F_* to select the parents of the next generation. In monoculture, direct and indirect genetic effects for yield can be estimated from a combination of yield records on plants, data on their position in the field (so that their neighbors are known) and pedigree or genome wide marker data (Muir, 2005; Cappa and Cantet, 2008; Silva et al., 2013). In this approach, knowledge of the mechanisms or traits underlying the competitive effects is not needed; instead, the full competitive effects for the traits of interest are estimated directly from the resulting phenotypes together with the genetic relationships between individuals in the population, using statistical mixed-model technology.

Extension of this statistical approach to intercropping is straightforward in principle. It merely requires extending the mixed model with an additional indirect genetic random effect due to the identities of the neighbors of the other crop. However, optimization of the design with respect to the spatial organization of families of each crop in relation to their neighbors will require careful consideration, to avoid confounding and to maximize precision of the resulting estimates of *G_D_* and *G_I_* of each of the two crops. The availability of genome-wide marker data should considerably increase the precision of these estimates, because it provides precise information on genetic relatedness between all individuals in the data. In cases where such data is not available, factorial designs are needed, in which each genotype of a species is tested in several mixtures with different genotypes of the other species.

### Analogy With Breeding for Hybrid Combining Ability

The analogy between hybrid breeding and intercrop breeding has already been drawn many times, in that both seek optimal combinations of genotypes. In hybrid breeding, the aim is to identify parental lines which, together, exhibit a good combining ability leading to heterosis in the *F*_1_ generation. In order to identify such parents, test crosses with a single or small number of tester lines are often performed (alternative approaches include a poly-cross or diallel; Acquaah, 2020). For intercrop breeding, using a single tester line of crop B when trialling crop A reduces the complexity to a level similar to that of a single-crop breeding program, providing a simple method to screen for “general mixing ability” or “general ecological combining ability” (Harper, 1964, 1967; Hill, 1990). However, such an approach would not yield sufficiently accurate information on the IGEs of the focal crop on the tester crop. Furthermore, specific interactions with the tester genotype would be included in the estimated genetic merit of individuals of the target crop (present in both direct and IGEs), which may bias breeding value estimates. As an alternative, a small set of tester lines selected for their contrasting phenotypes could be assembled or mixed to represent the range of possible cropping partners (Holland and Brummer, 1999). This could be a pragmatic and cost-effective strategy to begin with, although the specific choice of tester genotypes could potentially have a large influence on results. A highly competitive or dominant tester line may suppress genotypic differences in the target crop, while a weak tester may not provide sufficient inter-specific interaction (Hill, 1996). A recurrent selection scheme for the simultaneous improvement of two species was already proposed over 35years ago (Wright, 1985) and has been recently included in a simulation study that compared different selection strategies for intercrop performance (Sampoux et al., 2020). In this study, the bulked progenies of candidate lines from crop B were used as a tester for crop A and vice versa (Sampoux et al., 2020).

*F*_1_ hybrid breeding also distinguishes between general-combining and specific-combining abilities, with much focus on accurately estimating these parameters using phenotypic, pedigree, and genomic information. General mixing ability is the sum of the direct and IGEs, while specific mixing ability is the sum of the interaction terms between specific genotypes of both species (Forst et al., 2019; Haug et al., 2021). However, as pointed out earlier, in the context of a recurrent selection program for polygenic traits, specific combining effects are not inherited from one selection cycle to the next one.

### Randomization

Randomization is one of the central tenets of good experimental design. It helps guard against unwanted confounding between effects and non-experimental variables and underpins the assumption of independence of errors from ordinary linear models. However, intercrop trials can obstruct the process of randomization, since the regular patterns between alternating rows or strips are often by necessity non-random. In trials where the neighbor crop is one of the experimental factors, this factor cannot be randomly applied to the experimental units (e.g., sub-plots within strips). One possible solution is the use of spatial models, which attempt to correct for spatial trends in the analysis rather than at the design stage. When applied to data for a series of intercropping experiments looking at border effects, spatial models were found to improve the model fit in some but not all tested datasets (Knörzer et al., 2010). For plant breeding programs with relatively “simple” breeding objects – for example, finding the best genotype combination of maize and bean, a regular planting design need not overly bias the results if each recorded plot experiences a similar interaction environment; here, neighboring species is not an experimental factor of interest. However, the introduction of systematic biases (e.g., light interception patterns due to strip orientation) is often unavoidable, and therefore, careful planning of experiments is needed. If breeding is being performed to select a specific genotype that performs well with a wide range of other companion crops (in the broadest sense, as a target crop and good neighbor), then randomization issues become extremely pertinent. Simple designs are not always best in such situations (Connolly et al., 2001).

### Evolutionary Breeding

The idea of allowing natural selection to play a part in how a heterogeneous population develops has been termed “evolutionary breeding” (Suneson, 1956) and, although usually applied to intraspecific diversity, does fall under the wider topic of breeding for more genetically diverse systems. The possible benefits of such diverse populations are well documented, particularly under lower input conditions (Phillips and Wolfe, 2005; Dawson and Goldringer, 2012), providing a level of buffering against environmental variability. They also offer the possibility of developing local strains or farmer varieties through on-farm seed saving. Composite cross-populations, generated from a diverse panel of founder genotypes, provide a starting point for evolutionary breeding and have also featured in experiments aimed at developing varieties for intercropping (Allard and Adams, 1969; Hill, 1990). The potential of evolutionary breeding as a tool for intercrop breeding has been again recently highlighted (Annicchiarico et al., 2019), allowing component crop species to co-evolve over a number of generations. The authors did caution about its applicability for inbred crops, which may have limited evolutionary scope to improve complementarity traits (Annicchiarico et al., 2019). Many self-fertilizing species naturally have a low level of outcrossing, but a refinement to the original evolutionary breeding strategy was to introduce a certain proportion of male sterility in the population, promoting outcrossing and leading to hybrid seed production over multiple generations (Suneson, 1951, 1956; Phillips and Wolfe, 2005). Assuming that sufficient out-crossing occurs to produce a representative quantity of seed on male sterile plants, this would allow evolutionary progress (as opposed to dominance of a single genotype) to take place over practical time-scales.

For evolutionary biologists, competition effects in communities play a central role in so-called tragedies of the commons, where co-operation among a group of individuals is continually vulnerable to invasion from selfish individuals (Hardin, 1968; Gersani et al., 2001). In an agricultural context, the superior individual performance of dominant highly competitive individuals is often not reflected in the collective performance of such individuals when placed together in a field or in a pen (Weiner et al., 2017). Indeed, the process of domestication and artificial selection has often run contrary to natural evolutionary processes to avoid or circumvent such tragedies of the commons (Denison, 2012; Anten and Vermeulen, 2016; Montazeaud et al., 2020). On the one hand, evolutionary breeding may be vulnerable to potential tragedies of the commons. However, it could provide a complementary avenue to develop diverse and robust plant populations, particularly in the context of on-farm seed saving and farmer-engaged breeding efforts.

### Genetic Resources for Intercrop Breeding

Breeding is the exploitation of genetic variation for humankind’s benefit. It is an effort to both increase and decrease variation within the context of a single species (Louwaars, 2018; Schouten et al., 2019). Therefore, the issue of whether the genetic resources for improved intercrop performance are present in existing modern cultivars is of primary importance to intercrop breeding.

It is worth first examining whether existing genetic diversity within a crop species has demonstrated any functional purpose in crop mixtures. In natural systems, within-species genetic diversity is likely to play an important role in productivity (Hughes et al., 2008). In grassland systems, intraspecific diversity has been shown to result in positive biodiversity effects, for example in increased yield stability (Prieto et al., 2015). Fewer experiments have been performed in crop species, although meta-analyses of cereal performance (with a focus on wheat) have reported over-yielding to occur in crop mixtures (Kiær et al., 2009; Borg et al., 2018; Reiss and Drinkwater, 2018). Recent evidence suggests that there is a significant genotypic component in the ability of plant mixtures to over-yield (in this case, its domestication status: either wild or cultivated), tested over a range of important crop species (Chacón-Labella et al., 2019).

**Figure 3:**
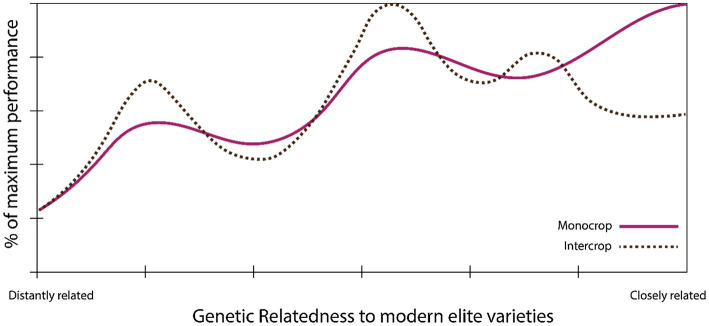
Theoretical performance landscape of modern varieties for intercropping. Modern elite varieties may not be optimal for intercropping, due, for example, to reduced partitioning of assimilates to seeds or reduced biodiversity effects when grown in mixtures (Chacón-Labella et al., 2019; Chen et al., 2021). Breeding efforts may have to break through local optima (dashed line) by accessing wider pools of genetic diversity in order to re-equip crops with features suited to intercropping.

Chacón-Labella et al. (2019) also found that biodiversity effects may have been reduced in the process of domestication. This suggests possible increases in intercrop performance could be achieved by re-diversifying the genetic basis of agricultural crops (Figure 3), although the performance gap between modern varieties, landraces, and crop wild relatives would require serious breeding attention.

Although not the only metric to judge relative performance, yield remains a central target of most breeding efforts, whether for monoculture or intercropping. It appears that modern cultivars may reduce the proportion of assimilates allocated to seed production when grown in mixtures, despite showing overall higher yields in both vegetative and reproductive plant parts in mixtures when compared to monoculture (Chen et al., 2021). As we do not know what theoretical limits exist regarding resource partitioning of crops grown in mixtures to seeds or other edible parts, it is too early to say whether breeding efforts could increase yield gains further, but the implication, particularly in the light of this recent evidence, is that it should be possible.

Studies have also shown differences in the root microbiome between wild ancestors and the cultivated progenitors, but again with a mixed pattern. For example, in barley (*Hordeum vulgare*), below-ground microbe communities were altered in small but significant ways depending on whether a modern cultivar, landrace, or wild accession was grown (Bulgarelli et al., 2015; Alegria Terrazas et al., 2020). For soya bean (Liu et al., 2019) and wheat (Valente et al., 2020), a more diverse microbiome was reported for wild ancestors than for crops, but for lettuce, domestication increased rhizobiome diversity (Cardinale et al., 2015). For maize, a history of 80years of breeding did not leave an imprint on the microbiome (Emmett et al., 2018). While these studies showed differences in species composition and diversity, linking such differences in microbiome functioning has still turned out to be elusive in most cases. Only Liu et al. (2019) showed that, despite taxonomic divergence in the microbiome of the wild ancestor and modern soya bean, there was functional convergence between both microbiome communities.

Overall, there is a need to assess whether the genetic resources currently available contain sufficient heritable variation for intercrop performance, and, if not, whether this could be increased by accessing wider gene-pools beyond that of modern elite germplasm. Without a systematic assessment of this, we risk making only marginal improvements in intercrop performance at great effort.

## Discussion

### A Powerful Troika: IGE, Plant Growth Models, and Genomic Prediction

In this paper, we have described two complementary breeding strategies for intercrop breeding: trait-based versus product-based. This dichotomy has also previously been recognized, where “trait-based” and “trait-blind” breeding approaches were identified (Gaba et al., 2015; Barot et al., 2017). Barot et al. (2017) proposed that these approaches be combined, using information on trait complementarity to perform an initial selection, after which a trait-blind strategy would select superior combinations. Here, we take a closer look at how these different strategies can complement each other.

Indirect genetic effect models come in two types: variance component models and trait-based models (McGlothlin and Brodie, 2009; Bijma, 2014). Variance component models do not model the IGE as a linear function of traits of the companion species, but instead partition the total genetic variance in the focal trait into a direct effect attributable to the focal individual and IGEs attributable to its social partners using linear models (e.g., Muir, 2005). They are empirically very powerful, but do not specify the causal traits and thus provide no knowledge of the underlying mechanisms. Trait-based models, in contrast, represent a functional approach that specify the IGE on an individual as a function of specific traits of its neighbors (Moore et al., 1997). Trait-based IGE models are a powerful approach when good prior information or a hypothesis on the traits underlying the IGE is available, particularly when phenotypes for these traits can be recorded precisely, but become statistically less tractable when multiple traits and reciprocal interactions are involved (Bijma, 2014).

To illustrate the two models, we compare the trait-based model of Moore et al. (1997) to the corresponding variance component model. Following Moore et al. (1997), considering interaction between two individuals, the value *z_i_* for trait *i* of the focal individual may be expressed as the sum of an additive genetic component of the focal individual, *a_i_*, a general (i.e., non-social) environmental component, *e_gi_*, and a component due to the values *z_j_* of each of *j*=1 to *n* traits of the partner,


$$z_{i}=a_{i}+e_{gi}+\overset{n}{\underset{j=1}{\sum }}\Psi_{ij}z_{j}^{\prime }$$

where the ′ indicates the social partner. Here, the $\Psi_{ij}$ is a path coefficient from trait *j* of the partner to trait *i* of the focal individual and the product $\Psi_{ij}z_{j}^{\prime }$ specifies the impact of trait *j* in the partner on the value of trait *i* in the focal individual. Hence, this model attributes indirect effects to specific traits (*j*) of the social partner. The corresponding variance component model is given by


$$z_{i}=A_{D_{i},\mathrm{focal}}+A_{I_{i},\mathrm{partner}}+e_{i}$$

where $A_{D_{i},\mathrm{focal}}$ is the (direct) genetic effect of the focal individual on its own value for trait *i* and $A_{I_{i},\mathrm{partner}}$ represents the full IGE of the partner on the value of trait *i* in the focal individual, without making reference to specific causal traits in the partner. Trait-based IGE models represent a functional approach with a focus on between-plant interactions and could therefore be complemented by FSP models. While variance component models disregard the functional traits underlying plant–plant interactions, such knowledge could considerably advance the precision of the phenotypes and thus the accuracy of selection. For example, FSP modeling coupled with information on phenotypic correlations could be used to determine which trait combinations optimize intercrop performance and whether such combinations are feasible (Picheny et al., 2017; van Eeuwijk et al., 2019).

**Figure 4:**
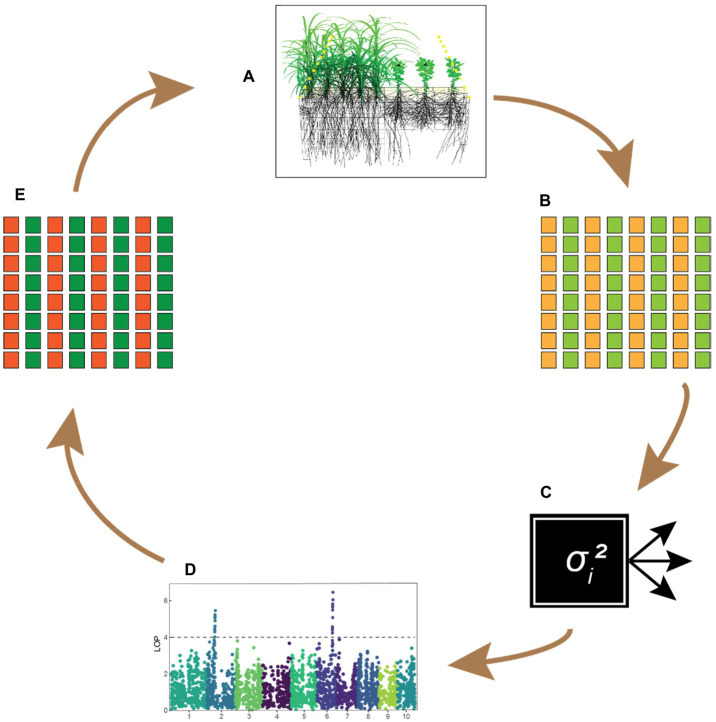
Theoretical framework integrating FSP models with a quantitative genetics approach for intercrop breeding. **(A)** Functional–structural plant models can be used to test crop combinations *in silico*, providing hypotheses for traits that improve crop complementarity. **(B)** Predicted traits are tested in practice using trait-based indirect genetic effect (IGE) models. **(C)** Variance-component models (shown here as a black box) determine whether the effects represent a meaningful proportion of the total genetic variance. **(D)** Genome-wide associations studies may reveal whether any major loci underlie differences in intercrop performance, to be used as fixed effects in a genomic prediction model. **(E)** Superior-performing genotypes are combined in further field trials, providing new data to update FSP models. This refinement step will lead to a new set of hypotheses on complementarity traits, renewing the cycle.

Moreover, statistical and functional models could be used as complementary approaches to identify the phenotypic traits functionally underlying the interactions (Figure 4). On the one hand, predictions based on functional models could be compared to empirical data to see whether their predictions match observed effects, in particular whether predictions from functional models agree with estimated genetic regression coefficients, and potentially also to identify new traits not (yet) present in the current gene pool (Figures 4A–C). On the other hand, variance component models can be used as a black-box tool to select populations for lower competitiveness (Figure 4C). Subsequently, the observed changes in functional traits provide information on which phenotypic traits underlie the competitive interactions, which may be used to improve FSP models (Figures 4D,E). In this approach, breeders let “the plants figure it out.” This approach may also lead to the identification of new traits that play an important role in plant–plant interactions and thus also has an exploratory function. Furthermore, the ability of plant growth models to simulate an extensive range of phenotypes without the normal constraints has the potential to predict novel phenotypes or phenotype combinations not yet encountered by breeders. Such traits could potentially provide breakthrough advances in intercrop breeding programs that might not have been otherwise achieved.

These traits may be included explicitly as predictors in a selection program using precision phenotyping (e.g., measured by sensors carried by unmanned arial vehicles or field robots, or using lab analyses). Recording many phenotypes at high precision is relatively costly, and breeders should utilize such information to the best possible extent. This is where genomic prediction (GP) can play a key role. With classical breeding for polygenic traits, the value of an individual phenotype is restricted to the individual itself and its close relatives. Hence, in a classical setting, precision phenotyping would need to be performed on “all” candidates for selection. With genomic prediction, however, information collected on a limited set of individuals can be utilized for a much broader set of candidates for selection that may be somewhat distantly related to the phenotyped individuals. In this way, GP could considerably increase the value of precision phenotypes, while removing the dependency on having complete phenotypic information before selection decisions can be made. Moreover, multivariate application of GP would give insight into the (genetic) relationships between the different traits involved in plant–plant interactions and could therefore inform FSP models with stochastic elements.

### Intercrop Breeding Without the Intercrop

Most breeding activities are currently performed in single-crop settings, reflecting the predominant monoculture agricultural paradigm. Although we have been considering specific breeding approaches for intercropping, we are assuming that IGEs are an important component in an intercrop system, not just in their magnitude but also in their potential correlation to direct genetic effects. However, this has yet to be firmly established for many important crop combinations and represents an important start for further research in this direction. This echoes the call to prioritize research into the linkage or correlation between “agronomic traits” and “interaction traits” (Litrico and Violle, 2015).

A high-input pure stand that discards data from border rows arguably provides a much more uniform environment than even a well-designed intercropping trial. In plant breeding, particularly in early-stage trials, the unit of selection is usually a single row or a small plot, which only loosely approximates the growing conditions of a large monoculture field. At later stages of a breeding program, plot sizes may increase as the number of genotypes to test decreases, but at this point many of the crucial early selections have already occurred. It is interesting to speculate that the necessity of selection procedures based on small plot performance (e.g., small seed lots, many genotypes to test, and limited space) may have inadvertently facilitated selection for intercrop performance or at least, to non-uniform competition effects from neighbors. However, these neighbors are usually of the same species as the target genotype. The literature on this topic tends to view such non-uniform effects as nuisance (Rebetzke et al., 2014) rather than as potentially beneficial for the long-term prospects of breeding for diversity.

If IGEs can be ignored, it would be preferable to continue to select in a more uniform pure stand than in an intercrop. A recent study on the application of genomic prediction for intercropping modeled a genetic correlation between monocrop and intercrop yield (Bančič et al., 2021) as the main parameter controlling the shared heritable information between a pure stand and mixed stand. Through simulation, it was found that the magnitude of this genetic correlation influences the optimum breeding strategy to apply (i.e., whether to include information from monocrop trials or not in a prediction model). The authors went on to argue that genotype x genotype interactions (which we understand to be another term for IGEs) will be minimized through the use of continuous complementary recurrent selection schemes (Hill, 1996; Bančič et al., 2021). However, it is not clear why heritable variation for G×G should tend to zero before that of direct genetic effects, nor whether this is a desirable strategy in the context of long-term genetic gain (Gorjanc et al., 2018; Vanavermaete et al., 2020).

Another approach to the question “do we need to breed for intercropping in an intercrop” has been to compare selection efficiencies between pure stands and mixed stands. Selection efficiency has previously been defined as $S=\frac{Y-A}{X-A}$ %, where *X* is the number of genotypes selected in the pure stand, *Y* is the number of pure-stand selected genotypes that were also selected in the intercrop, and *A* is the random expectation for *Y*, sampled from a binomial distribution (Hamblin and Zimmermann, 1986). This parameter has some advantages in that it says something about the reality of a running breeding program and the selective pressure being applied in a specific situation. However, it says nothing about the IGEs of the focal crop on its neighbors. Framing the issue as a genotype×cropping system interaction has also been used to test whether selection efforts for intercropping should be done in an intercrop system or not, depending on the level of significance of the interaction term (Gebeyehu et al., 2006). Again, this approach remains limited unless both direct and indirect genetic effects are considered. In many studies, there has been evidence of weak correlations between traits across genotypes evaluated in intercrop and monocrop systems (Zimmermann, 1996; Holland and Brummer, 1999), which at least provides a motivation to breeders to start testing their varieties under intercrop conditions. Indeed, some traits are simply not expressed in pure stands (in particular, the effect a plant has on its neighbors and vice versa) and cannot be evaluated without a mixed-crop setting.

## Concluding Remarks

We began this piece with agriculture at a crossroads. Diversified agriculture points a clear route toward more sustainable and productive systems. Although breeding for intercropping is by no means simple, it offers the possibility to re-align our crops with the cropping systems of the future, both above and below the ground. It is clear that breeding for intercropping will not become widespread without sufficient economic justification. Currently, research is underway to determine which crop combinations perform well together (not just in terms of yield, but also positive effects on bird and insect populations for example). There are also many well-established crop combinations that are used for intercropping worldwide (e.g., maize and bean) that provide well-tested models upon which to build. Once compatible cropping partners are known, the approaches described here can be used to estimate the magnitude of genetic variation for intercrop performance. Such knowledge, coupled with an increased uptake of diversified agriculture by farmers (perhaps incentivized for its positive impacts on biodiversity), will provide the breeding sector with a clear direction and justification. We are already witnessing a renewal of interest in the topic of intercrop breeding (not just in academia but in the wider breeding community) and anticipate further significant developments in this area in the coming years, in both theory and practice.

## Author Contributions

All authors listed have made a substantial, direct and intellectual contribution to the work, and approved it for publication.

## Funding

This work was partly financed through the Wageningen University & Research research programs “Connected Circularity” (project number KB-40-005-004) and “Breeding4Diversity” (KB-34-007-019). DA was partly funded by the research programme ‘Transition to ecology-based circular agriculture through the application of crop diversity’, a collaboration between Wageningen University & Research (WUR) and three private partners, with funding provided by the Topsector programme Agri & Food.

## Conflict of Interest

The authors declare that the research was conducted in the absence of any commercial or financial relationships that could be construed as a potential conflict of interest.

## Publisher’s Note

All claims expressed in this article are solely those of the authors and do not necessarily represent those of their affiliated organizations, or those of the publisher, the editors and the reviewers. Any product that may be evaluated in this article, or claim that may be made by its manufacturer, is not guaranteed or endorsed by the publisher.
